# The erosion of the US health-care workforce: silence is not an answer

**DOI:** 10.1016/j.lana.2025.101054

**Published:** 2025-03-14

**Authors:** The Lancet Regional Health – Americas

President Donald Trump has an agenda in his second term, and he is on it at full speed. Just over a month has passed since he took office, and many decisions that affect people's lives and health have been made. Between Jan 20 and Feb 25, he has issued 108 executive orders, more than any other recent president. After naming Elon Musk a special government employee and appointing him to lead the Department of Government Efficiency (DOGE), cuts have been made targeting the federal workforce and funds for humanitarian aid, such as USAID. This has significantly disrupted the US health-care workforce and spread uncertainty within the research community.

One of the latest executive orders is the termination of 1165 workers from the National Institution of Health (NIH) and a cut to limit indirect cost rates for all new and existing grants to 15%, impacting all research fields. Trump and Musk's partnership implies some of their aims and goals, which include diminishing the USA's role as a global leader in humanitarian aid and dismantling the workforce involved in research and health. These decisions, aligned with the America First discourse, are a mistake and will probably backfire. For example, the international aid not only benefits other countries but also protects the world from the spread of diseases and has historically worked as America's greatest soft power.

Being the largest US funding institution for research, the main goal of the NIH is to improve the health of the USA by conducting and supporting research. At the NIH, US$48 billion are allocated for medical research, of which 83% is designated to extramural research, 11% to support the research of 6000 NIH scientists, and 6% to support administrative and maintenance costs. Halting research funds and aggressively laying off more than a thousand probationary researchers has immediate and long-term effects, resulting in the loss of decades of research in the blink of an eye. Not only are ideas crushed, but also new treatments, drugs, and advances in research areas are severely hindered. These decisions affect US citizens and the rest of the world. For example, patients with terminal diseases seek clinical trials in the USA for alternative treatment, most of which are funded by the NIH. International projects can also be funded by the NIH, and several Central American and Caribbean countries have their core research activity dependent on external grants from the NIH and other US and European agencies.

The abrupt resignation of Dr Lawrence A Tabak, the number 2 official at the NIH, on Feb 11 is even more unsettling during this critical period. While still unconfirmed, his nominee for NIH director, Jay Bhattacharya, is a controversial scientist who critiqued the COVID-19 lockdown. Nominating someone who does not adhere to evidence-based science undermines the institution and jeopardises its integrity. Trump's decisions in these first months of his presidency are extremely problematic for the future of science and have a direct impact on people's lives. On Feb 14, more than 200,000 probationary federal workers of several different government institutions received a job termination letter without advance notice. The mass firing will result in high unemployment, impacting workers, their families, and their mental health. The remaining employees will also be under severe stress, being insecure about their current jobs and the future of their research. Will Trump terminate the positions or fill them in with his like-minded nominees? While the answer is unclear, laying off more than 220,000 workers could lead to a health sector employment crisis. If Trump decides to replace the workforce with like-minded allies, the scenario might be even worse.

By turning health and scientific institutions into agenda-driven entities, decisions pertaining to research topics, funding allocations, and public health policies might be influenced by political motives. Decisions should be guided by scientific evidence, and governments need to work aligned with scientists for the sake of people's right to health. While science is directly related to the society and subjected to political decisions, there must be a degree of independence. This is time to reunite, strategize, and act. Science and medicine evolve through the relentless efforts of the brightest minds dedicated to their fields. These individuals work tirelessly with the singular goal of saving and improving lives. There is no medicine without scientists, and there is no advancement without research. Dismantling the scientific workforce is a disastrous decision with far-reaching consequences for all. We must protect our scientists, respect their contributions, and trust their expertise. The scientific community must stand up, speak out, and advocate for those being silenced and disrespected.

These are worrisome times for the future of medical research in the USA and globally, and the research community needs to stay united and strong. The Lancet Group immediately responded to President Trump's executive order on the use of language related to sex and gender in research, reinstating our position on inclusive language, aligned with the International Committee of Medical Journal Editors. Four editorials were published across the group: *The Lancet* on Trump's decisions and their impact in the USA, *The Lancet HIV* on the impact of Trump's orders on people affected by HIV, *The Lancet Psychiatry* on the reflection on his current strategy and identification of which areas of mental health need the most breakthroughs, and *The Lancet Planetary Health* on the disappearance of US climate data.

At *The Lancet Regional Health—Americas* we stand with the research community and will continue to be a platform for equity, diversity, and inclusion and evidence-based science for the Americas region.

